# Organometallic small molecule kinase inhibitors – direct incorporation of Re and ^99m^Tc into Opaganib®†

**DOI:** 10.1039/d1cc03678e

**Published:** 2021-11-20

**Authors:** Raphael Lengacher, Youchao Wang, Henrik Braband, Olivier Blacque, Gilles Gasser, Roger Alberto

**Affiliations:** University of Zurich, Department of Chemistry Zurich Switzerland ariel@chem.uzh.ch; Chimie ParisTech, PSL University, CNRS, Institute of Chemistry for Life and Health Sciences, Laboratory for Inorganic Chemical Biology Paris F-75005 France

## Abstract

[(η5-Cp)Re^I^(CO)_3_] was incorporated into the kinase inhibitor Opaganib®. The resulting bioorganometallic complex showed a similar anti-cancer activity to Opaganib® against PC-3 cancer cells. The IC_50_ value for the kinase SK2 is 30x higher than that of Opaganib®. The ^99m^Tc homologue was synthesized, completing a matched-pair for molecular theranostics.

## Abstract 2

Replacing an adamantyl unit in the protein kinase inhibitor Opaganib® with an integrated [(η^5^-Cp)M(CO)_3_] (M = Re, ^99m^Tc) unit retains the lead's bioactivity and yields a true matched-pair pharmacomimetic.

## Graphical abstract

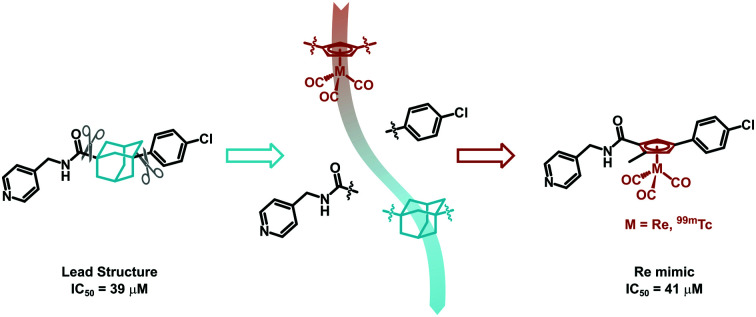

Small molecule kinase inhibitors (SMKIs) have emerged as highly relevant compounds in the clinical treatment of cancer and central nervous system (CNS) diseases.^1^ This is evidenced by no less than 28 SMKIs being approved by the FDA between 2001 and 2015.^2,3^

Incorporating a radioactive label into SMKIs allows for the tracking of the drug's distribution inside the body, enabling cancer diagnostics by positron emission tomography (PET) or single photon emission computed tomography (SPECT) imaging, depending on the isotope employed. Furthermore, an accurate quantification of kinase expression in tumour lesions *in vivo* accelerates drug discovery.^4^ A large number of radiolabelled SMKIs have been reported so far. Most of these are labelled with the PET radionuclides ^18^F and ^11^C since the non-radioactive isotopologues contain these elements in the lead structure.^5^ The monitoring of tyrosine kinase inhibition requires prolonged times for allowing sufficient accumulation of the radiolabelled inhibitor, thus, radioisotopes with a longer half-life time are favourable. A number of ^99m^Tc labelled SMKIs have been prepared, in which a chelator for coordinating Tc is appended to a SMKI.^6–10^ In this “pendent approach”, although the radiolabelled ^99m^Tc complex is spatially separated from the SMKI pharmacophore, the inclusion of an appended ^99m^Tc radiolabelled complex adversely affects pharmacokinetics and target receptor binding. Therefore, such ^99m^Tc-radiolabelled tracers cannot be used to image SMKI distribution and site-specific accumulation *in vivo*. Alternatively, in the integrated approach, the radiolabelled metal complex moiety comprises an integral chemical component of the pharmacophore, and its inclusion is important for receptor binding.^11,12^ The less the overall structure of the inhibitor changes, the less its pharmacology is affected. Examples are artificial amino acid mimics or integrated ferrocifen,^13–15^ which pioneered this field.

^99m^Tc cytectrenes ([(η^5^-Cp)^99m^Tc^I^(CO)_3_] complexes) have been employed as imaging agents mainly along the pendent but rarely the integrated approach.^20^ There are some previous examples for the integrated approach, *e.g.* the [(η^5^-C_5_H_4_-R)Re(CO)_3_] derivative of tamoxifen, a selective estrogen-receptor modulator by Jaouen and coworkers.^16^ Highly substituted rhenocene as ligands for the estrogen receptor have been presented by Katzenellenbogen and coworkers.^17^ The non-steriodal antiandrogen flutamide by Benny *et al.* is another example^18^ or Luyts macrocyclic peptides with integrated or pendent chelators.^21,22^ Some representative examples are given in Scheme 1. Not all of these examples describe both, the rhenium and the ^99m^Tc compounds, but they align with the concept of an integrated approach. The cytectrene moiety usually replaces a phenyl ring. With metal complex-derivatives of protein kinase inhibitors, Meggers and co-workers showed that higher affinities and selectivities as compared to the organic lead structures could be obtained.^19,23^Scheme 1d shows staurosporine and a derived ruthenium complex. The affinity of the latter for S6K1 was substantially higher, confirming that the metal complex is essential for inhibition. We herein report the synthesis of two organometallic analogues of the SMKI Opaganib. Opaganib® comprises a bulky adamantyl group (Fig. 1). We have designed our organometallic derivatives to retain both the pyridyl and chlorophenyl groups, while the cytectrene replaces the central adamantyl. In this way, the two terminal groups are separated from each other by three carbon atoms, as in the lead Opaganib® (Fig. 1).

**Scheme 1:**
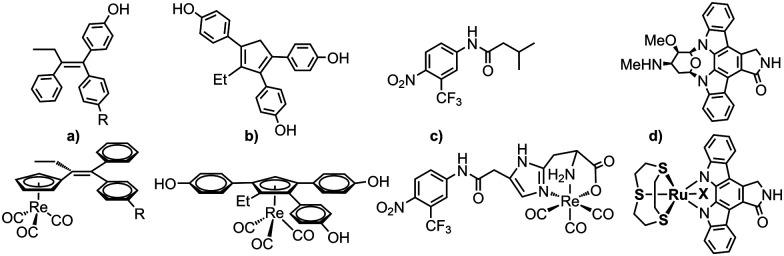
Examples for the integrated approach; tamoxifen and the [(η^5^-C_5_H_5_)Re(CO)_3_] analogue (a),^16^ a *de novo* estrogen receptor ligand (b),^17^ a flutamide in which the complex participates in binding (c)^18^ and a staurosporine kinase inhibitor (d).^19^

**Fig. 1:**
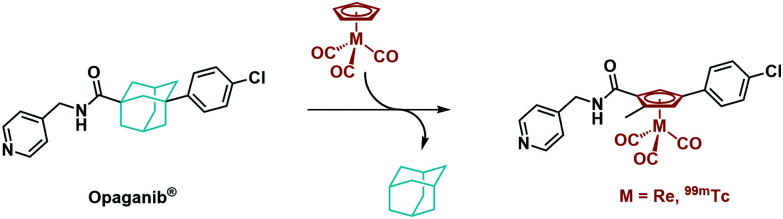
Conceptual replacement of an adamantyl moiety by a [(η^5^-Cp)M^I^(CO)_3_] (M = Re, ^99m^Tc) complex.

Opaganib®, also known as ABC294640, is an orally available, competitive and highly selective sphingosine kinase-2 (SK2) inhibitor (*K*_i_ = 9 μM).^24–26^ Opaganib® accumulates in tumours and induces apoptosis.^26^ This accumulation makes Opaganib® an interesting lead for the development of radio imaging agents. The adamantyl moiety was replaced by a [(η^5^-Cp)M^I^(CO)_3_] (M = Re, ^99m^Tc) complex (Fig. 1), which showed very similar IC_50_ values towards PC-3 cells, exemplifying the validity of the integrated approach.

The formation of the core cyclopentadiene (Cp) synthesis followed a report of Hatanaka *et al.*^27^ We applied this procedure to the synthesis of various Cp-based building blocks over the recent years,^15,28,29^ however, always with alkylated but not with arylated cyclopentadienes. By reacting 2-bromo-4-chloroacetophenone (1) with the Wittig salt 2^27^ under basic conditions, Cp 3 was obtained in 22% yield. We emphasize that this procedure is applicable to other bromo-acetyl aromatics of which numerous precursors for pharmaceuticals are commercially available; this method can thus principally be extended to other pharmaceuticals. Upon reaction of [Re_2_(CO)_10_] with 3 at high temperature, the Re complex 4 was obtained in 72% yield (see ESI†). The ester in compound 4 was hydrolysed to its carboxylate 5 with aqueous NaOH (1 M) at high temperatures in 58% yield. Subsequent amide bond formation with 4-methylaminepyridine gave the [(η^5^-Cp)Re^I^(CO)_3_] incorporating Opaganib® analogue 6 in good yield (67%, Scheme 2).

**Scheme 2:**
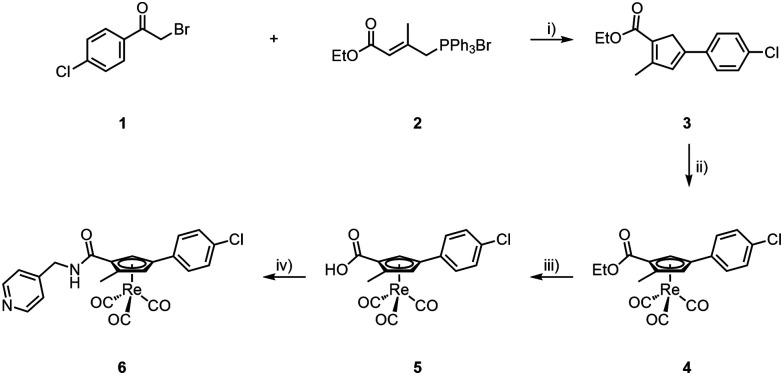
Synthesis of 6; (i) NaHCO_3_, CH_2_Cl_2_/H_2_O, r.t., 24 h, 22%; (ii) [Re_2_(CO)_10_], *o*-xylene, μ-wave, 220 °C, 45 min, 72%; (iii) NaOH, MeOH/H_2_O, μ-wave, 120 °C, 15 min, 58%; (iv) 4-aminomethylpyridine, HOBt, EDC, DIPEA, DMF, r.t., 17 h, 67%.

Single crystals were obtained for compounds 3, 4, and 6. Crystallographic details for 3 and 4 can be found in the ESI.† Single crystals of 6 (Fig. 2) were obtained by slow evaporation of a H_2_O/MeCN solution after being spiked with a small amount of NH_4_PF_6_. The distance between the Cp centroid and Re1 was calculated to be 1.958 Å. Re–CO bond distances are all within the same range with 1.907(4) Å (Re1–C1), 1.912(4) Å (Re1–C2), and 1.923(3) Å (Re1–C3) and Re–C–O bond angles of 175.2(3)° (Re1–C1–O1), 175.7(3)° (Re1–C2–O2), and 178.97(3)° (Re1–C3–O3). The structural properties are in line with similar complexes known in the literature (Scheme 2).^15,30–36^

**Fig. 2:**
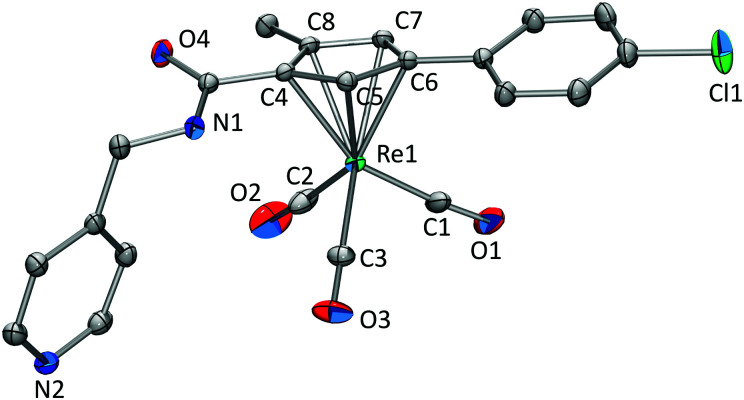
Displacement ellipsoid representation of 6. Hydrogens are omitted for clarity. Thermal ellipsoids represent 35% probability.

**Scheme 3:**
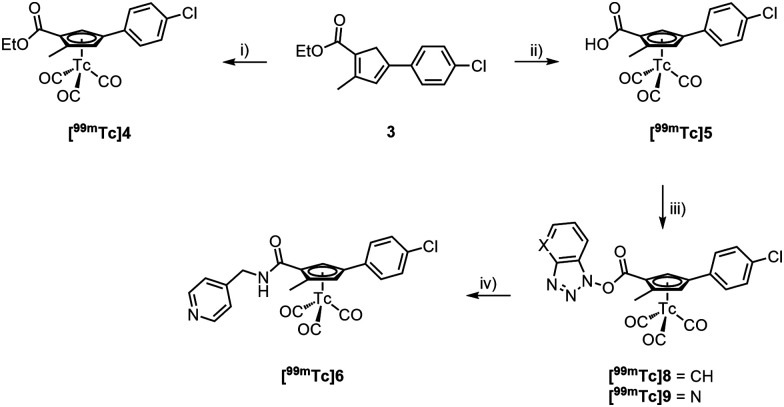
Synthesis of [^99m^Tc]6; (i) [^99m^Tc(H_2_O)_3_(CO)_3_], EtOH/H_2_O (pH = 12), μ-wave, 100 °C, 60 min, 90% RCP; (ii) [^99m^Tc(H_2_O)_3_(CO)_3_], EtOH/H_2_O (pH = 12), μ-wave, 110 °C, 30 min, 80% RCP; (iii) HOBt/HABt, CH_2_Cl_2_/MeCN, r.t., 30 min; (iv) 4-aminomethylpyridine, r.t., 30 min, 99% RCP.

We attempted to prepare the pure cyclopentadiene analogue of Opaganib® for activity comparison with the lead and for a direct labelling with the *fac*-[^99m^Tc(CO)_3_]^+^ core. However, this was unsuccessful as 3 could not be hydrolysed. Most likely, the cyclopentadiene is deprotonated under these alkaline conditions. The resulting negative charge is delocalized, including the ester group, which leads to a fulvene-like anion. In this form, the ester carbon is inaccessible for nucleophilic attack. Approaches for acidic hydrolysis were equally unsuccessful. Thus, for preparing the homologue [^99m^Tc]6, a post-labelling functionalization procedure had to be employed.^37^ Post-labelling modifications are less desired for routine application since they rely on multi-step radiosynthesis, However, for conceptual assessments it is still an option.^20^ Accordingly, 3 was reacted with [^99m^Tc(H_2_O)_3_(CO)_3_]^+^ (7) in an EtOH/H_2_O solution. Fulvene formation is inhibited in [^99m^Tc]4 after the formation of the cytectrenes and the ester group hydrolyses easily depending on the labelling conditions, [^99m^Tc]4 or [^99m^Tc]5 form in high radiochemical purity (90% RCP, resp. 80% RCP) in one step (Scheme 3).

Along peptide bond formation chemistry, [^99m^Tc]5 was activated with DCC and HOBt or HOAt in a CH_2_Cl_2_/MeCN mixture at r.t., forming the activated ester [^99m^Tc]8/[^99m^Tc]9 within 30 min. Its formation was monitored by radio-HPLC. Addition of excess 4-aminomethylpyridine for 30 min at r.t. afforded [^99m^Tc]6 with 99% RCP after HPLC purification (Fig. 3). The lipophilicity of both compounds 6 (*R*_t_ = 21.82 min) and Opaganib® (*R*_t_ = 22.90 min) is remarkably similar although, 6 is slightly more hydrophilic.

**Fig. 3:**
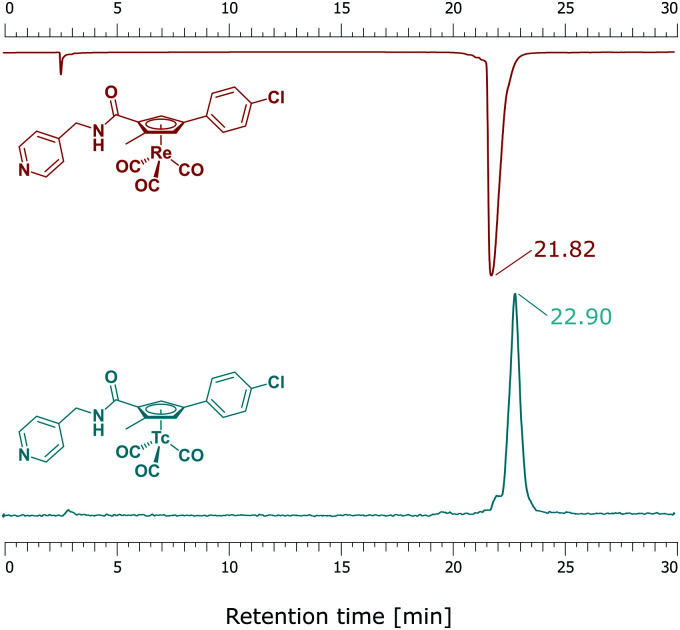
Coinjection of 6 (top) and [^99m^Tc]6 (bottom).

**Fig. 4:**
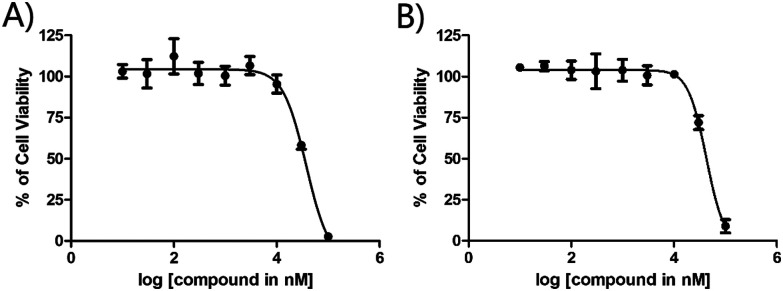
EC_50_ values of complexes incubated with PC3 cell line (left: 6; right: Opaganib®).

The cytotoxicity of 6 and Opaganib® towards PC3 cancer cell line (human caucasian prostate adenocarcinoma cells) and non-cancerous RPE1 cell line (retinal pigment epithelium cells) was investigated using a well-known fluorometric cell viability assay (Resazurin).^38^ Cisplatin was used as a control since it is a well-known metal-based anticancer drug. As shown in Table 1, Opaganib® displayed a moderate toxicity on both PC3 and RPE1 cell lines, with EC_50_ values of *ca.* 37 μM. As hypothesized, the EC_50_ values for 6 are similar to the ones of Opaganib® on both cell lines, demonstrating that 6 induces similar cytotoxicity to Opaganib® (Fig. 4). To furthermore confirm that 6 competitively binds to SK2, IC_50_ values of Opaganib and 6 towards SK2 were determined. Whereas a *K*_i_ value for Opaganib is available (*vide supra*), no IC_50_ has been reported. We found that Opaganib has an IC_50_ towards SK2 of 15.8 ± 3.0 μM and 6 a surprisingly low one of 0.4 ± 0.05 μM (ESI†), *ca.* 30x lower than Opaganib®. This is in agreement with other findings in which bioorganometallic analogues of pharmaceuticals displayed higher selectivities and affinities for receptors.^39–41^

**Table 1:** Cytotoxicity of Opaganib® and 6 in comparison with cisplatin

Compound	EC_50_ ± S.D.^a^ (μM)	
	PC3	RPE1
Opaganib®	37.8 ± 2.9	37.5 ± 2.0
6	41.4 ± 2.4	47.1 ± 2.7
Cisplatin	6.2 ± 0.9	29.0 ± 2.2

a Standard deviation.

In conclusion, cold Re and radioactive ^99m^Tc were both successfully incorporated into the lead structure of the SMKI Opaganib®, representing the first examples of organometallic SMKI analogues. Furthermore, [^99m^Tc]6 represents a ^99m^Tc radiolabelled SMKI following a truly integrated approach. 6 possess similar activity to Opaganib® *versus* PC-3 cancer cells, corroborating the validity of the concept presented in this article. Compound 6 exhibits a 30x higher affinity for SK2 compared with Opaganib, exemplifying that the integration of a metal complex into a pharmaceutical does not necessarily lead to loss of activity. The post-labelling modification procedure for the ^99m^Tc homologue of 6 was established but is not ideal for direct application. Preparation of cyclopentadienes carrying two different functionalities is not routine. The presented synthetic concept still represents a general procedure towards other, multi-functional cytectrene complexes with rhenium and ^99m^Tc. The search for alternative routes towards other cyclopentadiene analogues is currently ongoing.

This work was financially supported by an ERC Consolidator Grant PhotoMedMet to G. G. (GA 681679) and has received support under the program *Investissements d’Avenir* launched by the French Government and implemented by the ANR with the reference ANR-10-IDEX-0001-02 PSL (G. G.). Y. W. thanks the China Scholarship Council for financial support. The authors recognize financial support and the contribution of the analytical services of the chemistry department at the University of Zurich.

## Conflicts of interest

The authors declare no conflicts of interest.

## Supplementary Material

CC-057-D1CC03678E-s001

CC-057-D1CC03678E-s002
