# Supplementary material for: Organometallic small molecule kinase inhibitors – direct incorporation of Re and 99mTc into Opaganib®
Source: Chem Commun (Camb). 2021 Nov 20;57(98):13349–52. doi: 10.1039/d1cc03678e (PMC8658909; doi:10.1039/d1cc03678e)
Supplement: CC-057-D1CC03678E-s001 [file CC-057-D1CC03678E-s001.pdf]

Electronic Supplementary Material (ESI) for ChemComm.
This journal is © The Royal Society of Chemistry 2021

Supporting Information

Organometallic Small Molecule Kinase Inhibitors – Direct Incorporation of Re and ^{99m}Tc into Opaganib®

Raphael Lengacher‡, Youchao Wang, Henrik Braband, Olivier Blacque, Gilles Gasser, and Roger Alberto*

Department of Chemistry, University of Zürich, Winterthurerstrasse 190, 8057 Zürich, Switzerland

Chimie ParisTech, PSL University, CNRS, Institute of Chemistry for Life and Health Sciences, Laboratory for Inorganic Chemical Biology, F-75005 Paris, France.

1 Table of Contents

1 General Methods 2
2 Synthesis 4
3 Biological Data 7
4 Radiochemistry 9
5 Spectra 11
6 Crystallographic Data 20

1 General Methods

Materials and Techniques: Unless otherwise stated, all chemicals were of reagent grade or higher, obtained from commercial sources and used without further purification. Sodium boranocarbonate was a gift from *Mallinckrodt Medical B.V.* (The Netherlands). Solvents for reactions were of p.a. grade or distilled prior to their use; H_2O was bi-distilled. Deuterated NMR-solvents were purchased from *Armar Chemicals* or *Cambridge Isotope Laboratories, Inc.* (UK). Reactions were carried out using standard *Schlenk* techniques in oven-dried (120 °C) glass equipment and monitored for completion by analyzing a small sample (after suitable workup) by TLC, UPLC or UPLC-ESI-MS. Evaporation of the solvents *in vacuo* was done with the rotary evaporator. Normal phase column chromatography was carried out with *Merck* silica gel *60* (40–63 µm) with the indicated solvent system. Microwave assisted reactions were carried out in an *Anton Paar, Monowave 200* (Re-reactions) or a *Biotage Initiator* (^{99m}Tc-reactions) microwave.

NMR spectra: *Bruker AV2-400* (400 MHz) or *Bruker AV2-500* (500 MHz); in deuterated solvents at 298 K; chemical shifts (δ) in ppm relative to residual solvent resonances ($CDCl_3$ ^{1}H: δ 7.26, ^{13}C: δ 77.16); coupling constants (J) in Hz. Signal assignments are based on coupling constants, increment calculations and/or 2D-NMR experiments.

IR spectra: *SpectrumTwo FT-IR Spectrometer* (*Perkin–Elmer*) equipped with a *Specac Golden Gate^{TM} ATR* (attenuated total reflection) accessory; applied as neat samples; 1/λ in cm^{-1}.

HR-ESI-MS: *QExactive* (*Thermo Fisher Scientific*, Bremen, Germany) equipped with a heated ESI source connected to a *Dionex Ultimate 3000* UPLC system. Samples dissolved in MeOH, MeOH/CH_2Cl_2 3:1, MeOH/H_2O 1:1, DMSO/H_2O 1:10, or H_2O at ca. 50 µg mL^{-1}; injection of 1 µL on-flow with an XRS auto-sampler (*CTC*, Zwingen, Switzerland)(mobile phase: MeOH + 0.1% HCOOH or CH_3CN/H_2O (2:8) + 0.1% HCOOH; flow rate 120 µL mL^{-1}); ion source parameters: spray voltage 3.0 kV, capillary temperature 280 °C, sheath gas 30 L min^{-1}, s-lens RF level 55.0; aux gas temperature 250 °C; full scan MS in alternating (+)/(−)-ESI mode; mass ranges 80–1’200, 133–2’000, or 200–3’000 amu; resolution (full width half-maximum) 70’000; automatic gain control (AGC) target 3.00 10^6; maximum allowed ion transfer time (IT) 30 ms; mass calibration <2 ppm accuracy for *m/z* 130.06619–1621.96509 in (+)-ESI with *Pierce*® ESI calibration solutions (*Thermo Fisher Scientific*, Rockford, USA); lock masses: ubiquitous erucamide (*m/z* 338.34174, (+)-ESI).

UPLC-ESI-MS: *Waters Acquity* UPLC System coupled to a *Bruker Daltonics HCT^{TM}* ESI-MS, using an *Acquity UPLC BEH* C18 1.7 µm (2.1 x 50 mm) column. UPLC solvents were formic acid (0.1% in millipore water) (solvent A) and acetonitrile UPLC grade (solvent B). Applied UPLC gradient: 0–0.5 min: 95% A, 5% B; 0.5–4.0 min: linear gradient from 95% A, 5% B to 0% A, 100% B; 4.0–5.0 min: 0% A, 100% B. The flow rate was 0.6 mL min^{-1}. Detection was performed at 250 and 480 nm (DAD).

Preparative HPLC: *Shimadzu* system (CBM-40 system controller, SPD-40 UV-VIS detector, LC-20AO preparative liquid chromatograph, FCV-200AL prep quaternary valve), using a *Dr. Maisch Reprosil* C18 100-7 (40 x 250 mm) column. HPLC solvents were water (with 0.1% trifluoroacetic acid) (solvent A) and HPLC grade acetonitrile (with 0.1% trifluoroacetic acid)

(solvent B)The flow rate was 40 mL min^{-1}. Detection was performed at between 230 and 270 nm.

Elemental Analysis: Elemental Analysis (EA) measurements were performed on a LecoCHNS-932 elemental analyzer.

Radioactive materials: $Na[^{99m}TcO_4]$ in 0.9% saline was eluted from a $^{99}Mo/^{99m}Tc$ *Ultratechnekow FM* generator purchased from *b. e. imaging AG* (Switzerland).

HPLC analyses of ^{99m}Tc complexes: *Merck Hitachi Chromaster 5160* pump coupled to a *Merck Hitachi Chromaster 5430* diode array detector and a radiodetector. UV-Vis detection was performed at 250 nm. The detection of radioactive ^{99m}Tc complexes was performed with a *Berthold FlowStar LB 514* radiodetector equipped with a *BGO-X* cell. Separations were achieved on a *Macherey-Nagel NUCLEOSIL®* C18 5 µm, 100 Å (250 × 3 mm) column. HPLC solvents were HPLC grade acetonitrile (solvent A) and trifluoroacetic acid (0.1% in Millipore water) (solvent B). Applied HPLC gradient: 0–3 min: 0% A, 100% B; 3–3.1 min: 0–25% A, 100–75% B; 3.1–9 min: 25% A, 75% B; 9–9.1 min: 25–34% A, 75–66% B; 9.1–18 min: 34–100% A, 66–0% B; 18–25 min: 100% A, 0% B; 25–25.1 min: 100–0% A, 0–100% B; 25.1–30 min: 0% A, 100% B. The flow rate was 0.5 mL min^{-1}.

Crystal structure determination: Single-crystal X-ray diffraction data for compounds **3**, **4** and **6** were collected at 160(1) K on Rigaku OD diffractometers: a XtaLAB Synergy, Dualflex, Pilatus 200K diffractometer for **6** and a SuperNova/Atlas area-detector diffractometer for **3** and **4**, both diffractometers using the Cu K_α radiation (λ = 1.54184 Å) from a micro-focus X-ray source and an Oxford Instruments cryojet cooler. The selected suitable single crystals wer mounted using polybutene oil on a flexible loop fixed on a goniometer head and immediately transferred to the diffractometer. Pre-experiment, data collection, data reduction and analytical absorption correction[1] were performed with the program suite *CrysAlisPro*.[2] Using *Olex2*,[3] the structures were solved with the *SHELXT*[4] small molecule structure solution program and refined with the *SHELXL2018/3* program package[5] by full-matrix least-squares minimization on F^2. *PLATON*[6] was used to check the result of the X-ray analyses. CCDC numbers 2094782 for **4**, 2094783 for **3**, and 2094784 for **6** contain the supplementary crystallographic data for these compounds, and can be obtained free of charge from the Cambridge Crystallographic Data Centre via www.ccdc.cam.ac.uk/data_request/cif.

2 Synthesis

Synthesis of **3**

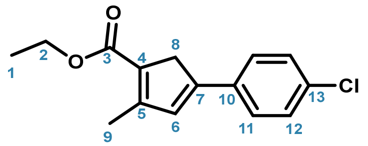

A round bottom flask (500 mL) equipped with a rubber septa was charged with **2** (4.69 g, 10 mmol, 1 eq) and CH_2Cl_2 (100 mL). The solution was overlaid with sat. aq. $NaHCO_3$ (100 mL) and stirred vigorously resulting in a colour change from colourless to bright yellow. 2-bromo-4-chloroacetophenone (2.34 g, 10 mmol, 1 eq) was added and the biphasic solution was stirred for 24 h at r.t. during which the colour turned dark red. The phases were separated, and the aqueous phase was extracted with CH_2Cl_2 (2×30 mL). The combined organic phases were washed with aq. HCl (1 M, 150 mL), dried over $MgSO_4$ and concentrated *in vacuo*. The resulting material was dry loaded onto silica gel and purified by column chromatography (silica gel, hexane/EtOAc 5:1). The title compound **3** (589.7 mg, 2.24 mmol, 22%) was obtained as an off-white solid.

^{1}H NMR ($CDCl_3$, 500 MHz): δ (ppm) = 7.47 (td, *J* = 8.5, 1.9 Hz, 2 H, H_{12}), 7.31 (td, *J* = 8.5, 1.9 Hz, 2 H, H_{11}), 6.74 (s, 1 H, H_6), 4.25 (q, *J* = 7.1 Hz, 2 H, H_2), 3.68 (d, *J* = 1.8 Hz, 2 H, H_8), 2.39 (t, *J* = 2.4 Hz, 3 H, H_9), 1.34 (t, *J* = 7.1 Hz, 3 H, H_1).

^{13}C NMR ($CDCl_3$, 126 MHz): δ (ppm) = 165.02 (C_q, 1 C, C_3), 155.60 (C_q, 1 C, C_5), 149.11 (C_q, 1 C, C_7), 133.7 (C_q, 1 C, C_{13}), 133.4 (C_q, 1 C, C_{10}), 132.4 (CH_1, 1 C, C_6), 128.9 (CH_1, 2 C, C_{11}), 128.2 (C_q, 1 C, C_4), 126.8 (CH_1, 2 C, C_{12}), 59.6 (CH_2, 1 C, C_2), 42.1 (CH_2, 1 C, C_8), 15.5 (CH_3, 1 C, C_9), 14.5 (CH_3, 1 C, C_1).

HR-ESI-MS: $[M+H]^+$ = calc. m/z 263.08336, found: m/z 263.08333 (0.08 Δppm).

IR (neat) ν [cm^{-1}]: 3660, 3441, 2983, 2258, 2025, 1932, 1727, 1592, 1492, 1445, 1402, 1371, 1258, 1092, 1013, 909, 830, 766.

Synthesis of **4**

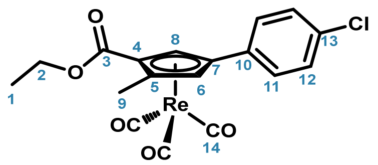

An *Anton Paar* G30 MW vial was charged with **3** (246 mg, 0.93 mmol, 1 eq), [$Re_2(CO)_{10}$] (291 mg, 0.47 mmol, 0.5 eq), and *o*-xylene (11 mL). The reaction mixture was stirred at 220°C for 45 min in the microwave, providing a brown solution. The solvent was removed under N_2-stream. The crude product was suspended in MeCN/H_2O (9:1) and filtered over Celite®. The filter cake was dissolved in CH_2Cl_2 and filtered. CH_2Cl_2 was removed under N_2-flow. The title compound **4** (356 mg, 0.67 mmol, 72%) was obtained as a grey solid.

^{1}H NMR ($CDCl_3$, 500 MHz): δ (ppm) = 7.31 (s, 4 H, H_{11} & H_{12}), 6.23 (d, *J* = 2.1 Hz, 1 H, H_8), 5.65 (d, *J* = 1.9 Hz, 1 H, H_6), 4.38-5.24 (m, 2 H, H_2), 2.60 (s, 3 H, H_9), 1.35 (t, *J* = 7.2 Hz, 3 H, H_1).

^{13}C NMR ($CDCl_3$, 126 MHz): δ (ppm) = 195.3 (C_q, 3 C, C_{14}), 165.2 (C_q, 1 C, C_3), 135.3 (C_q, 1 C, C1$_3$), 130.3 (C_q, 1 C, C_{10}), 129.7 (CH_1, 2 C, $C_{11/12}$), 128.0 (CH_1, 2 C, $C_{12/11}$), 111.9 (C_q, 1 C, C_4), 105.7 (C_q, 1 C, C_7), 86.0 (C_q, 1 C, C_5), 84.6 (CH_1, 1 C, C_8), 83.5 (CH_1, 1 C, C_6), 61.8 (CH_2, 1 C, C_2), 14.8 (CH_3, 1 C, C_1), 14.5 (CH_3, 1 C, C_9).

HR-ESI-MS: $[M+H]^+$ = calc. m/z 532.00827, found: m/z 532.00818 (0.16 Δppm).

IR (neat) ν [cm^{-1}]: 3102, 2924, 2023, 1920, 1700, 1515, 1474, 1439, 1418, 1312, 1240, 1199, 1160, 1083, 1014, 904, 833.

Synthesis of **5**

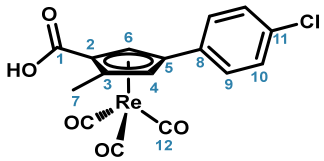

An *Anton Paar* G10 MW vial was charged with **4** (41 mg, 77.4 µmol, 1 eq), MeOH (1.4°mL), and aq. NaOH (1 M, 774 µL, 10 eq). The reaction mixture was stirred at 120°C for 15 min in the microwave. Aq. HCl (1 M, 1 mL) was added, resulting in immediate precipitation. The Precipitate was filtered off, dissolved in CH_2Cl_2 and filtered over Celite®. CH_2Cl_2 was removed under N_2-flow. The title compound **5** (22.8 mg, 45.2 µmol, 58%) was obtained as a colourless solid. The product was used without further purification in subsequent steps.

UPLC-ESI-MS: R_t = 3.1 min, $[M-H]^-$ = calc. m/z 502.97, found: m/z 502.92.

Synthesis of **6**

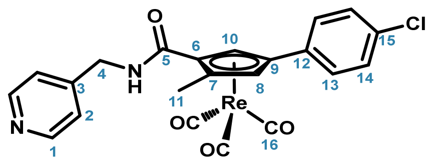

A 20 mL vial was charged with **5** (22.8 mg, 0.045 mmol, 1eq), 4-aminomethylpyridine (9.1 µL, 0.09 mmol, 2 eq), HOBt (14 mg, 0.1 mmol, 2.2 eq), and DMF (2.6 mL). The solution was stirred for 10 min at r.t.. Then, EDC (19 mg, 0.1 mmol, 2.2 eq) and DIPEA (28 µL, 0.2 mmol, 4.4 eq) were added and the resulting solution was stirred for 17 h at r.t.. The solvent was removed under N_2-stream and the crude material was dissolved in MeCN (2.04 mL) 0.1% TFA in H_2O (1.36 mL) and purified by preparative HPLC. The title compound **[6]TFA** (21.5 mg, 30.3 µmol, 67%) was obtained as a light brown solid.

^{1}H NMR ($CDCl_3$, 500 MHz): δ (ppm) = 8.55 (d, *J* = 4.6 Hz, 2 H, H_1), 8.16 (t, *J* = 5.3 Hz, 2 H, NH), 7.81 (d, *J* = 5.1 Hz, 2 H, H_2), 7.31 (s, 4 H, H_7 & H_8), 6.50 (d, *J* = 1.7 Hz, 1 H, H_{10}), 5.68 (d, *J* = 1.5 Hz, 1 H, H_8), 4.96 (dd, *J* = 16.9, 6.4 Hz, 1 H, H_{4a}), 4.51 (dd, *J* = 16.9, 4.7 Hz, 1 H, H_{4b}), 2.63 (s, 3 H, H_{11}).

^{13}C NMR ($CDCl_3$, 126 MHz): δ (ppm) = 193.4 (C_q, 3 C, C_{16}), 165.0 (C_q, 1 C, C_{14}), 160.5 (C_q, 1 C, C_3), 141.1 (CH_1, 2 C, C_1), 134.9 (C_q, 1 C, C_{15}), 129.4 (C_q, 1 C, C_6), 129.2 (CH_1, 2 C, $C_{13/14}$), 127.4 (CH_1, 2 C, $C_{14/13}$), 125.3 (CH_1, 2 C, C_2), 111.2 (C_q, 1 C, C_7), 105.4 (C_q, 1 C, C_{12}), 88.2 (C_q, 1 C, C_9), 83.3 (CH_1, 1 C, C_8), 81.4 (CH_1, 1 C, C_{10}), 42.6 (CH_2, 1 C, C_4), 14.1 (CH_3, 1 C, C_{11}).

HR-ESI-MS: $[M+H]^+$ = calc. m/z 595.04289, found: m/z 595.04274 (-0.25 Δppm).

EA: **[6]TFA·H_2O** calc. C, 39.70; H, 2.64; N, 3.86; found: C 39.11±0.237; H, 2.453±0.0147; N, 3.590±0.0193.

IR (neat) ν [cm^{-1}]: 3662, 3094, 2971, 2901, 2023, 1925, 1668, 1508, 1406, 1394, 1382, 1250, 1198, 1140, 1066, 1057, 879, 831.

3 Biological Data

Cell culture

Human Caucasian prostate adenocarcinoma (PC3) cells were cultured using F12K media and Retinal pigment epithelium (RPE1) cells using DMEM/F-12 medium supplemented with 10% FBS and 1% Penstrep. The cells were cultivated and maintained in a cell culture incubator at 37 °C with 5% CO_2 atmosphere. Before an experiment, the cells were passaged three times.

Cytotoxicity

The cytotoxicity of the compounds was assessed by measuring the cell viability using a fluorometric resazurin assay. The cultivated cells were seeded in sextuplicate in 96 well plates with a density of 4000 cells per well in 100 µL of media. After 24 h, the medium was removed and the cells were treated with increasing concentrations of the compound diluted in cell media achieving a total volume of 100 µL. Dilutions for Opaganib®, **6**, and Cisplatin (as a comparison) were prepared as follows: 10 mM stock in DMSO for Opaganib®, **6** and 1.5 mM stock in media for Cisplatin. These solutions were further diluted to 100 µM. After 48 h incubation, the media was replaced with fresh media containing resazurin with a final concentration of 0.2 mg/mL. After 4 h of incubation at 37 °C, the fluorescence signal of resorufin product was measured (λ_{ex}: 540 nm and λ_{em}: 590 nm) in a Tecan microplate Reader. EC_{50} values were then calculated using GraphPad Prism software. Each experiment was performed in triplicate and an average IC_{50} value (in µM) was reported with a standard deviation.

SK2 Inhibition

The natural substrate sphingosine is phosphorylated by the kinase SK2. For this process, ATP is converted to ADP. The rate of conversion is competitively inhibited in a concentration dependent manner by e.g. Opaganib or compound **6**. The ADP formation is quantified spectroscopically with the ADP-Glo™ Kinase Assay from Promega after fixed time points.[7] In short, SK2 phosphorylates sphingosine in the presence of the inhibitor. After 40 min, the reaction is stopped by the ADP-Glo reagent and excess ATP is removed. The kinase detection reagent is then added which converts the remaining ADP into ATP. The luciferase reaction turns ATP into light emission, which is recorded as the signal.

The SK2 inhibition experiments were performed by the International Centre for Kinase Profiling, MRC PPU Reagents and Services, School of Life Sciences, University of Dundee, Dow Street, Dundee, DD1 5EH, United Kingdom, using the luminescent ADP detection assay ADP-Glo^{TM} of Promega, which measures inhibition as %age (ATP-to-ADP conversion). Inhibition was recorded in triplicates for Opaganib, obtained from Selleck Chemicals and for compound **6**. Each data point was measured in duplicates and the IC50 values are the average of three determinations (Tables S1 and S2)

Experimental conditions: Sphingosine Kinase 2 (diluted in 100 mM Tris pH = 7.5, 400 mM KCl, 10 mM $MgCl_2$, 2 mM EDTA) is assayed in a total volume of 20 ul containing 0.02 mM Sphingosine and Opaganib or compound **6** in the respective concentrations (Tables S1 and S2) and at apparent ATP K_m values. The enzyme is assayed for 60 min after which 20 ul of ADP-Glo^{TM} reagent is added to stop the kinase reaction. 10 µL of Kinase Detection Reagent was added and luminescence output was read following 45–60 min incubation at room temperature with a PerkinElmer Envision instrument for 1 sec/well.

This assay follows the guidelines given by the manufacturer as described in ref 7.

Table S1. Competitive SK2 inhibition data for compound **6** vs. Sphingosine

Conc. 6 µM)	Conc. 6 (nM)	Log [6]	% SK2 activity	% SK2 activity	mean	s.d.
**0,003**	3	0,477121	101	101	101	0
**0,01**	10	1,000000	91	94	93	2
**0,03**	30	1,477121	84	85	84	1
**0,1**	100	2,000000	77	79	78	2
**0,3**	300	2,477121	58	60	59	1
**1**	1000	3,000000	36	35	35	1
**3**	3000	3,477121	28	27	27	1
**10**	10000	4,000000	15	18	16	2
**30**	30000	4,477121	8	7	7	0
**100**	100000	5,000000	3	3	3	1

Table S2. Competitive SK2 inhibition data for Opaganib® vs. Sphingosine

Conc. Opaganib (µM)	Conc. Opaganib (nM)	log[Opaganib]	% SK2 activity	% SK2 activity	mean	s.d.
**0,003**	3	0,477121	101	106	103	4
**0,01**	10	1,000000	103	101	102	2
**0,03**	30	1,477121	97	105	101	6
**0,1**	100	2,000000	100	95	98	3
**0,3**	300	2,477121	98	100	99	1
**1**	1000	3,000000	97	99	98	1
**3**	3000	3,477121	85	89	87	3
**10**	10000	4,000000	62	58	60	3
**30**	30000	4,477121	39	44	42	4
**100**	100000	5,000000	16	5	10	8

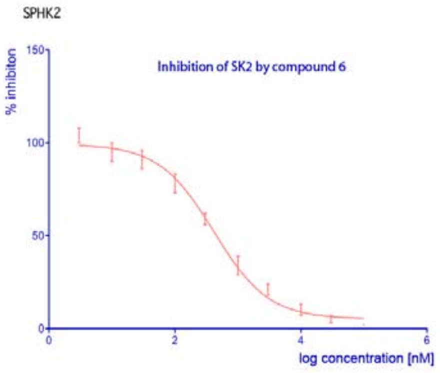

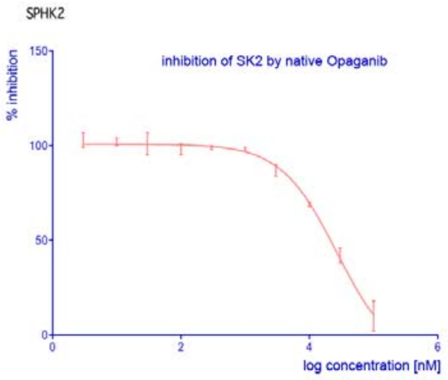

Figure S1. Graphical representation of SK2 inhibition by compound **6** (left) and Opaganib® (right)

4 Radiochemistry

Synthesis of **[^{99m}Tc(H_2O)$_3$(CO)$_3$] (7)**

A microwave vial was charged with the *isolink kit* chemicals sodium boranocarbonate (4 mg, 39 μmol), sodium tartrate dihydrate (7 mg, 30 μmol) and sodium tetraborate decahydrate (7 mg, 19 μmol). The vial was sealed and flushed with N_2 for 30 min. [^{99m}TcO_4]$^-$ eluate (2 mL) was added and the solution was heated in the microwave to 100 °C for 7 min. Unreacted sodium boranocarbonate was neutralized by dropwise addition of 1 M HCl to pH = 1. Then, the pH of the solution was adjusted to 12 by dropwise addition of 1 M NaOH.

Synthesis of **[^{99m}Tc]4**

A microwave vial was charged with 0.5 mL of a 5 mM solution of **3** in CH_2Cl_2. The vial was sealed and flushed with N_2 until no solvent remained in the vial. EtOH (0.5 mL) and freshly prepared [^{99m}Tc(H_2O)$_3$(CO)$_3$] solution (0.5 mL) were added and the solution was heated in the microwave to 100 °C for 60 min. Complex **[^{99m}Tc]4** was obtained in 37% RCP. Purification with HPLC delivered **[^{99m}Tc]4** in 90% RCP.

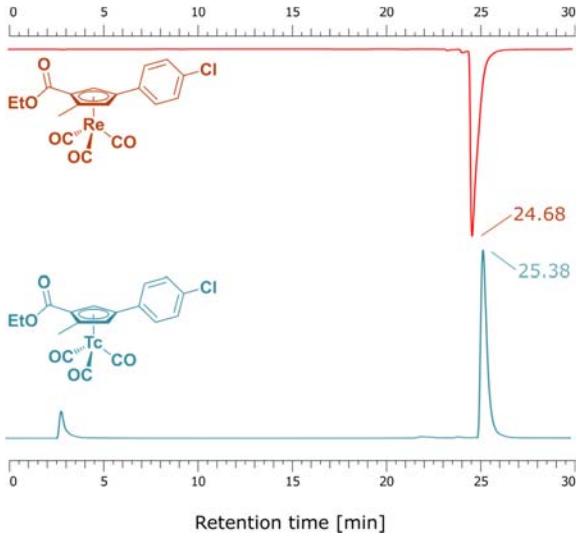

Figure S2: Coinjection of **4** with **[^{99m}Tc]4**.

Synthesis of **[^{99m}Tc]5**

A microwave vial was charged with 0.5 mL of a 5 mM solution of **3** in CH_2Cl_2. The vial was sealed and flushed with N_2 until no solvent remained in the vial. Sodium dodecyl sulfate (4 mg) was added and the vial was again flushed with N_2 for 30 min. EtOH (0.5 mL) and freshly prepared [^{99m}Tc(H_2O)$_3$(CO)$_3$] solution (0.5 mL) were added and the solution was heated in the microwave to 110 °C for 30 min, delivering **[^{99m}Tc]5** in 80% RCP.

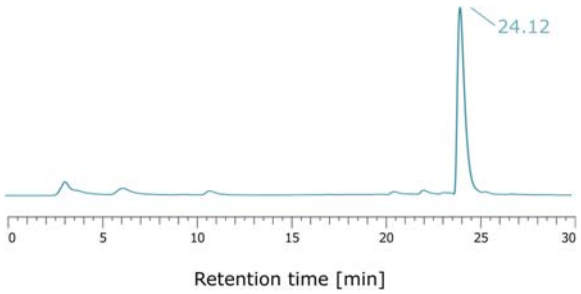

Figure S3: Radio HPLC trace of **[^{99m}Tc]5**.

Synthesis of **[^{99m}Tc]6**

The solution containing **[^{99m}Tc]5** was concentrated to dryness under N_2-stream. HOBt (3 mg) and DCC (3 mg) were added in a CH_2Cl_2/MeCN mixture (1:1, 1 mL) and the resulting solution was stirred for 30 min at r.t., resulting in the formation of the HOBt activated ester **[^{99m}Tc]8**. If HOAt is used instead of HOBt, **[^{99m}Tc]9** is formed instead of **[^{99m}Tc]8** (figure S4). Then, 4-aminomethylpyridine (0.1 mL) was added and the solution was again stirred for 30 min at r.t. which lead to the formation of **[^{99m}Tc]6** in 85% RCP. Purification with HPLC delivered **[^{99m}Tc]6** in 99% RCP as shown in Figure 3 of the manuscript.

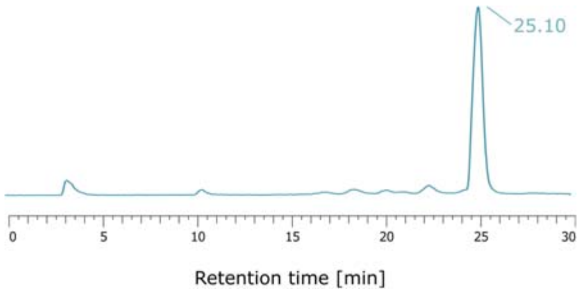

Figure S4: Radio HPLC trace of **[^{99m}Tc]9** 30 min after addition of HOAt.

5 Spectra

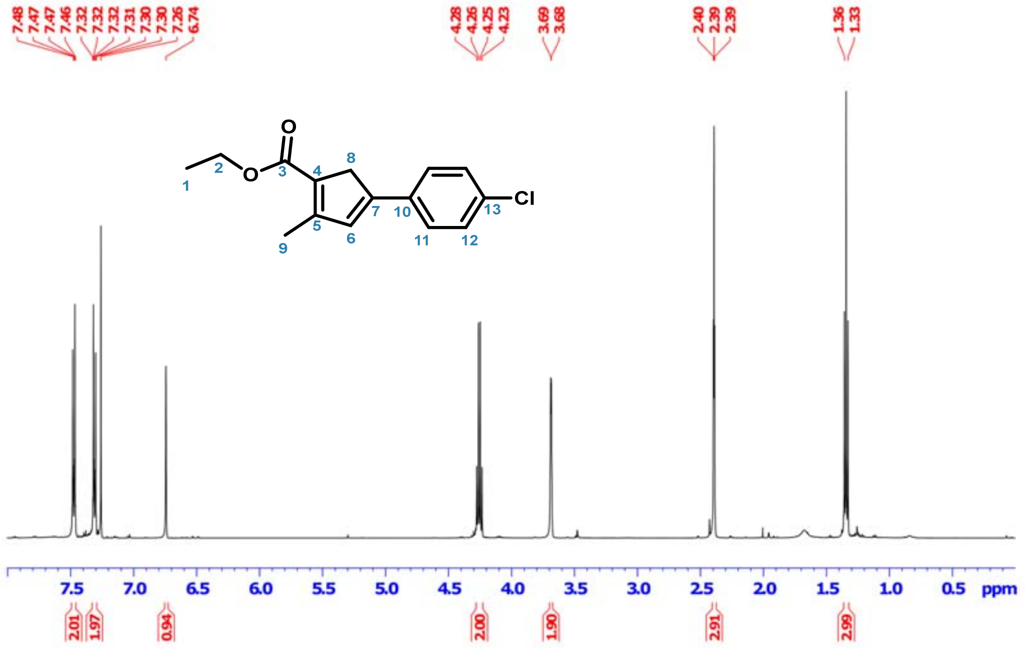

Figure S5: ^{1}H NMR of **3** in $CDCl_3$ (292 K).

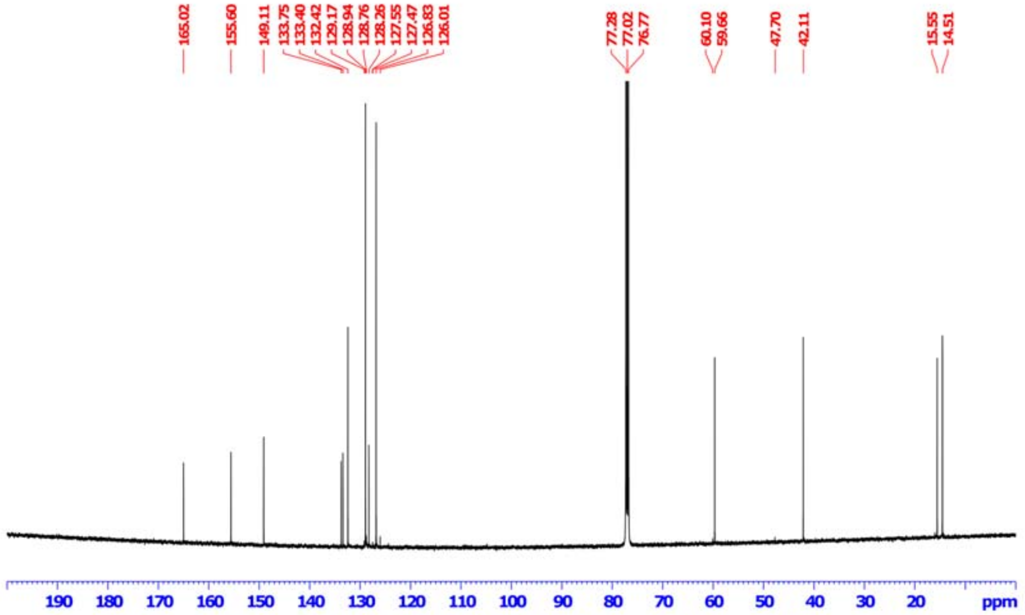

Figure S6: ^{13}C NMR of **3** in $CDCl_3$ (292 K).

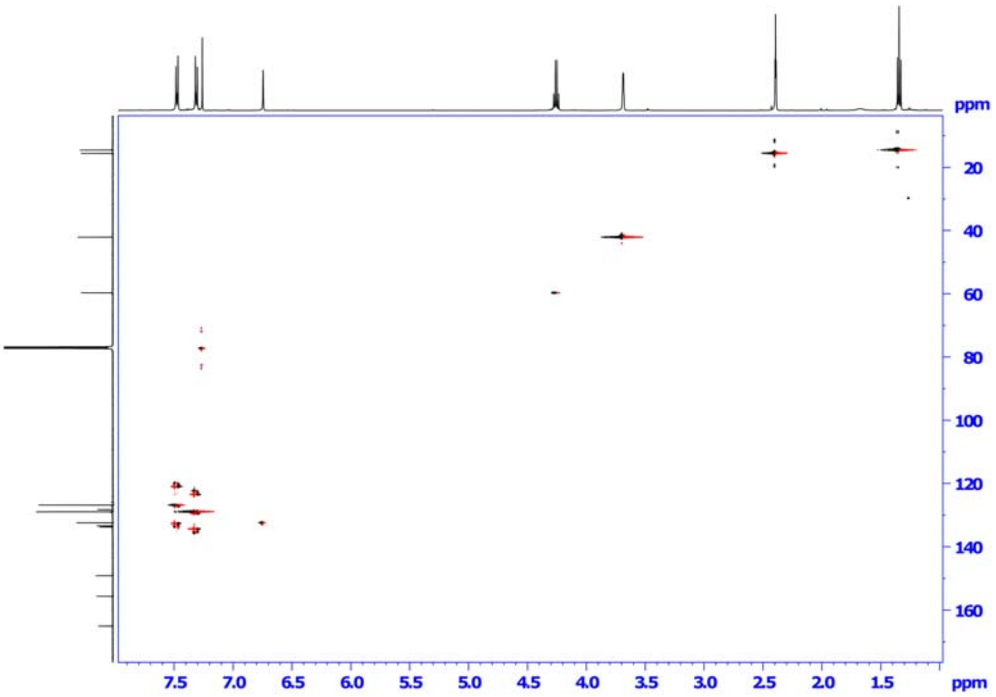

Figure S7: HSQC of **3** in $CDCl_3$ (292 K).

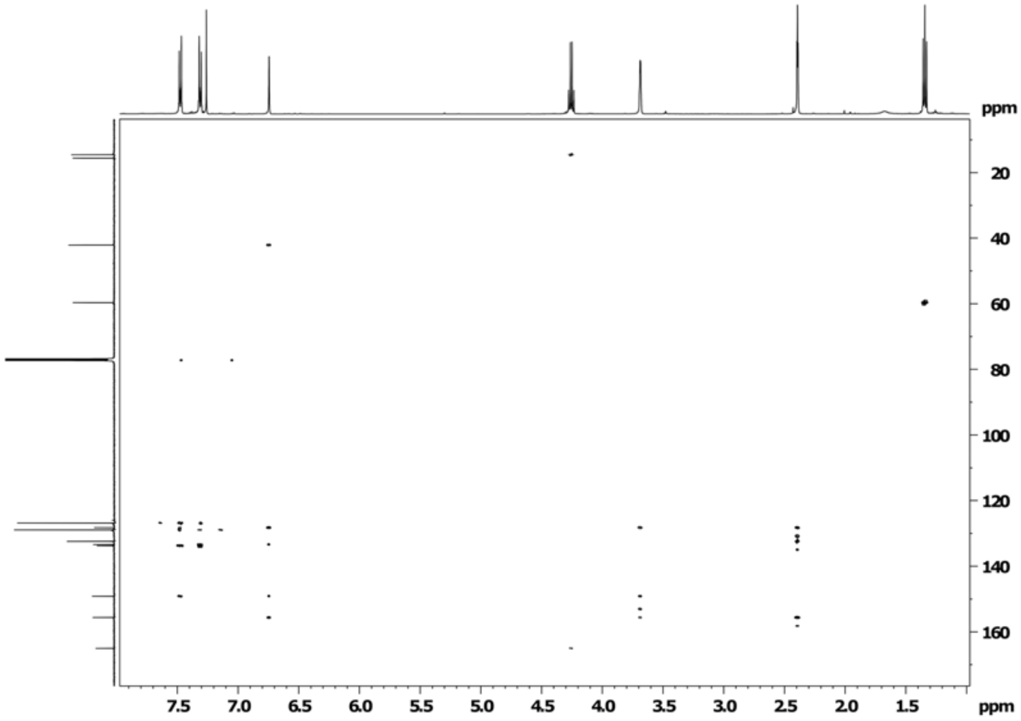

Figure S8: HMBC of **3** in $CDCl_3$ (292 K).

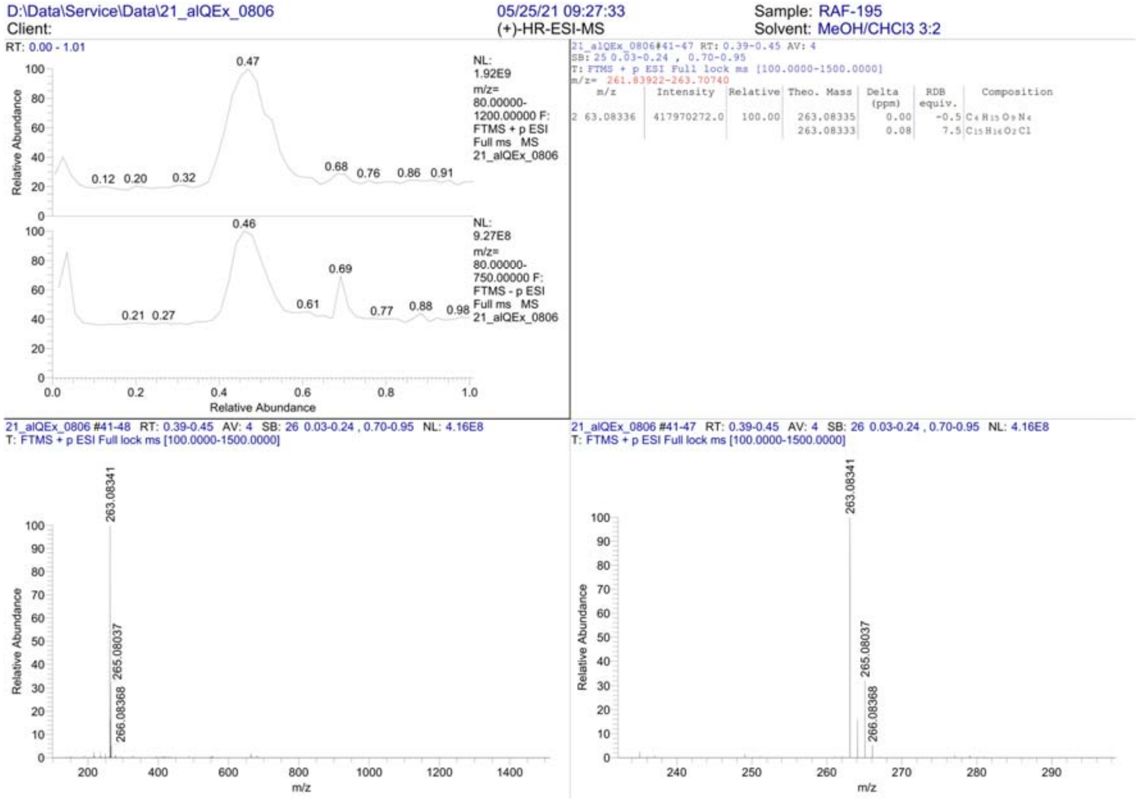

Figure S9: HR-ESI-MS of **3**.

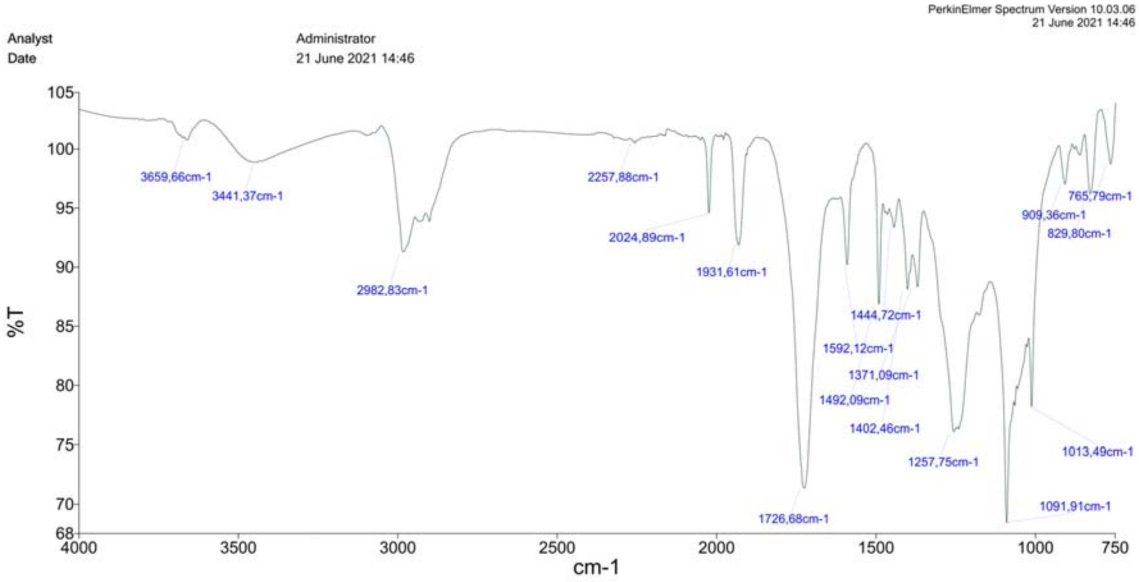

Figure S10: FT-IR of **3**.

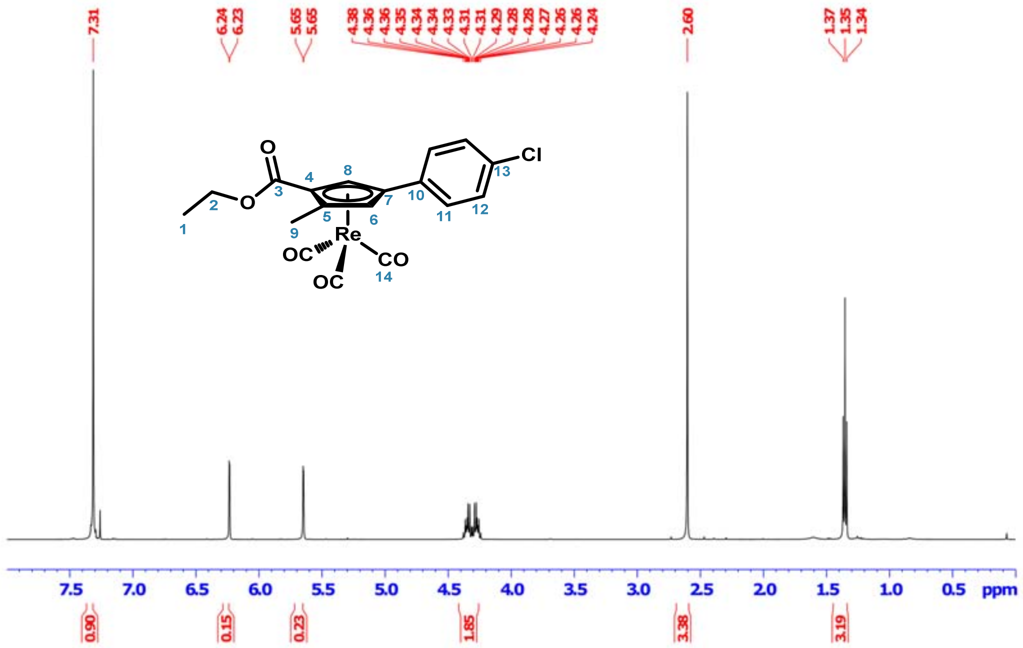

Figure S11: ^{1}H NMR of **4** in $CDCl_3$ (292 K).

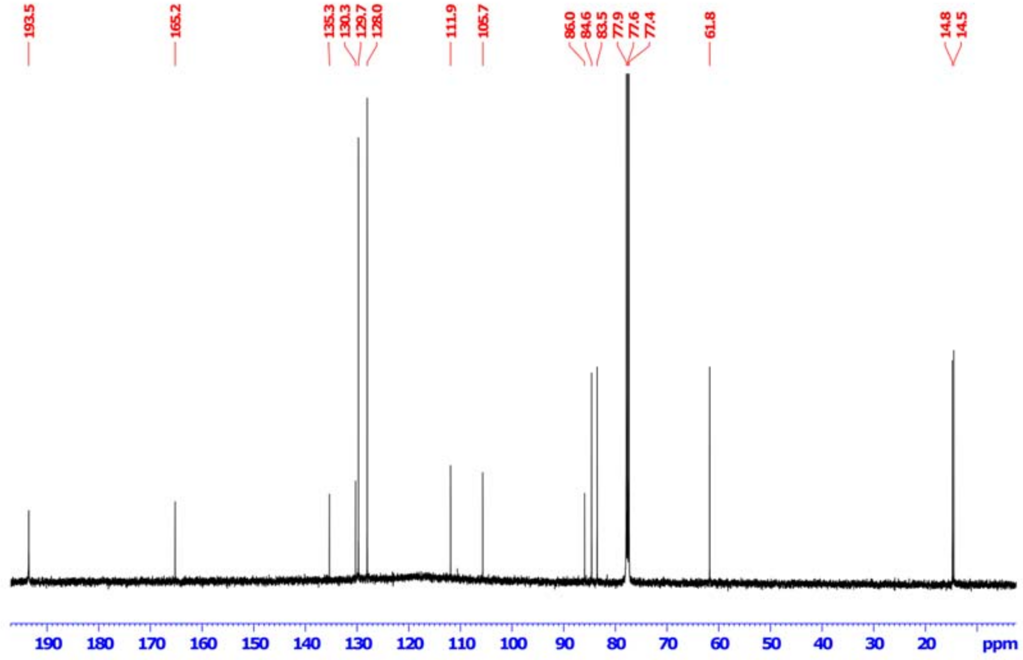

Figure S12: ^{13}C NMR of **4** in $CDCl_3$ (292 K).

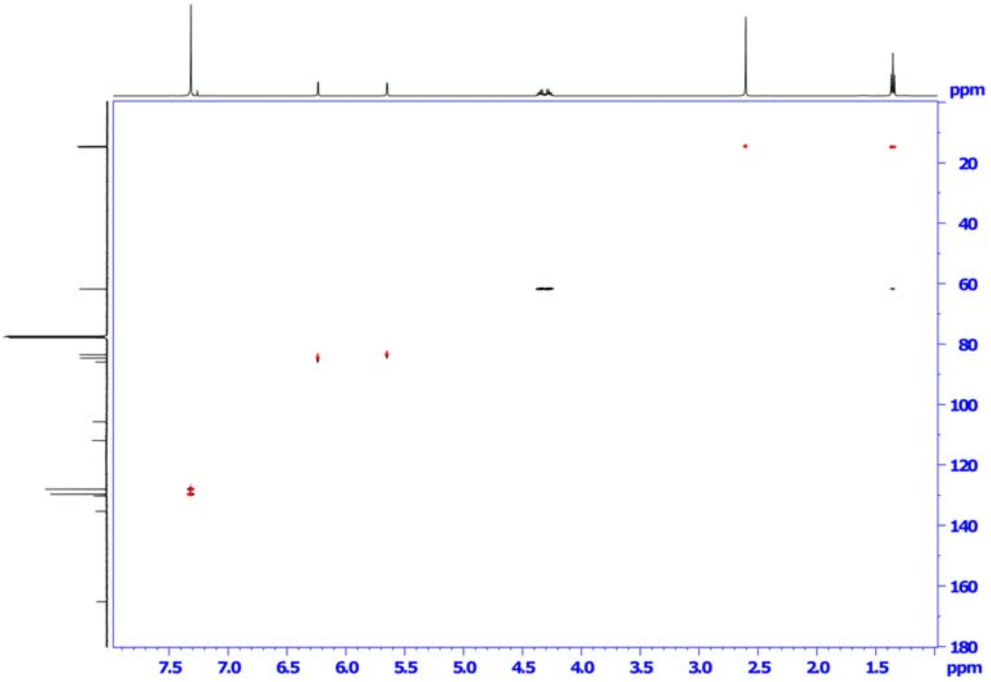

Figure S13: HSQC of **4** in $CDCl_3$ (292 K).

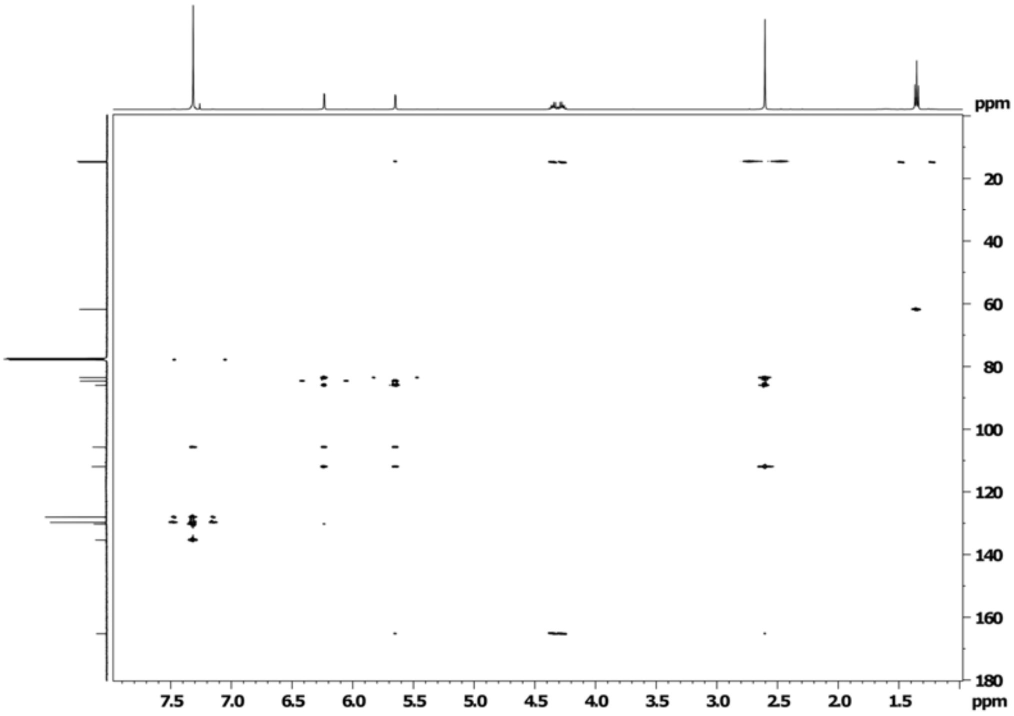

Figure S14: HMBC of **4** in $CDCl_3$ (292 K).

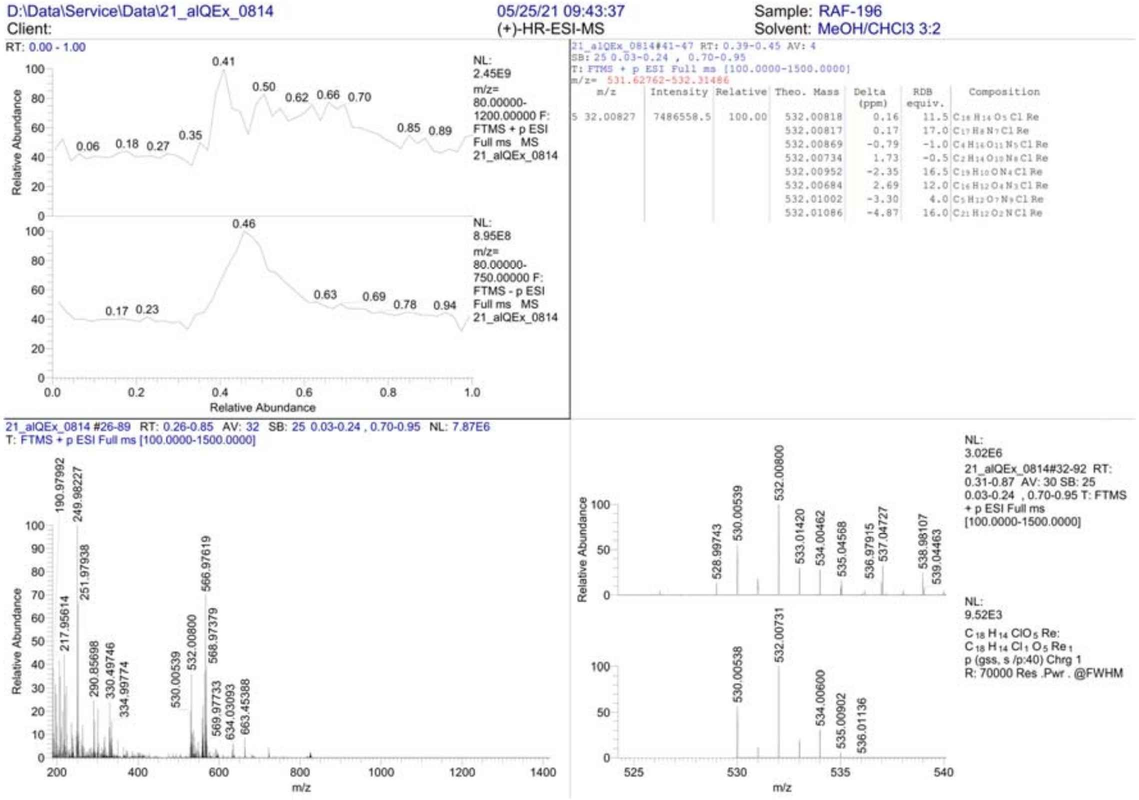

Figure S15: HR-ESI-MS of **4**.

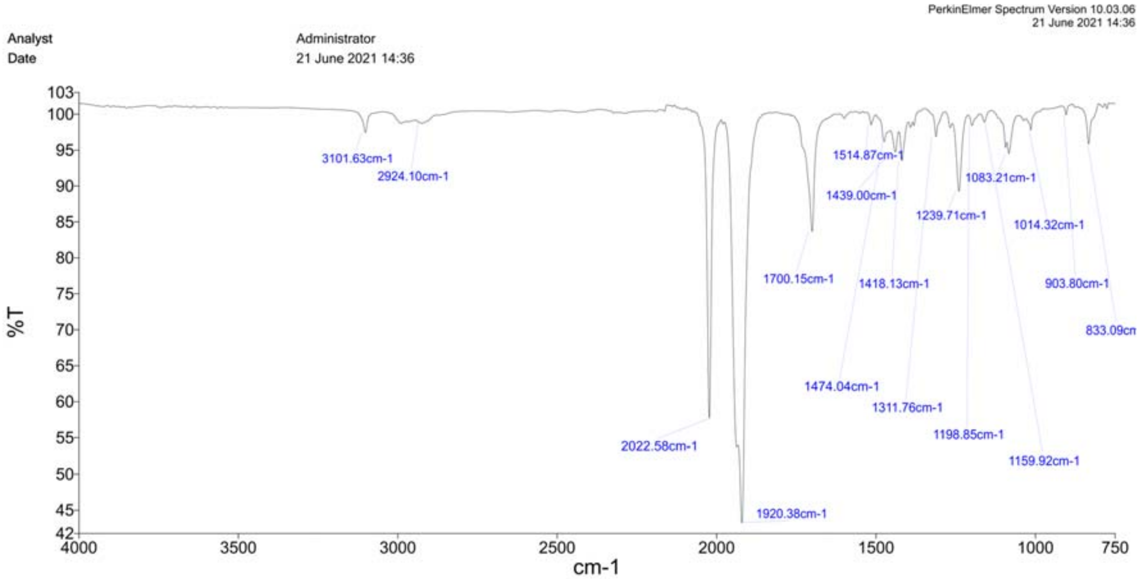

Figure S16: FT-IR of **4**.

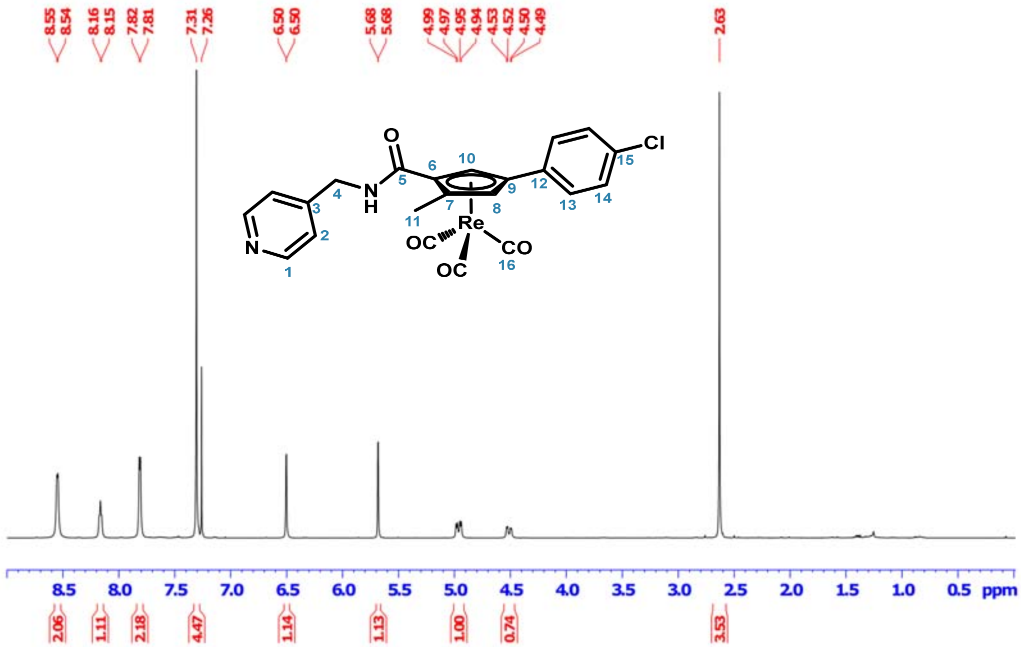

Figure S17: ^{1}H NMR of **6** in $CDCl_3$ (292 K).

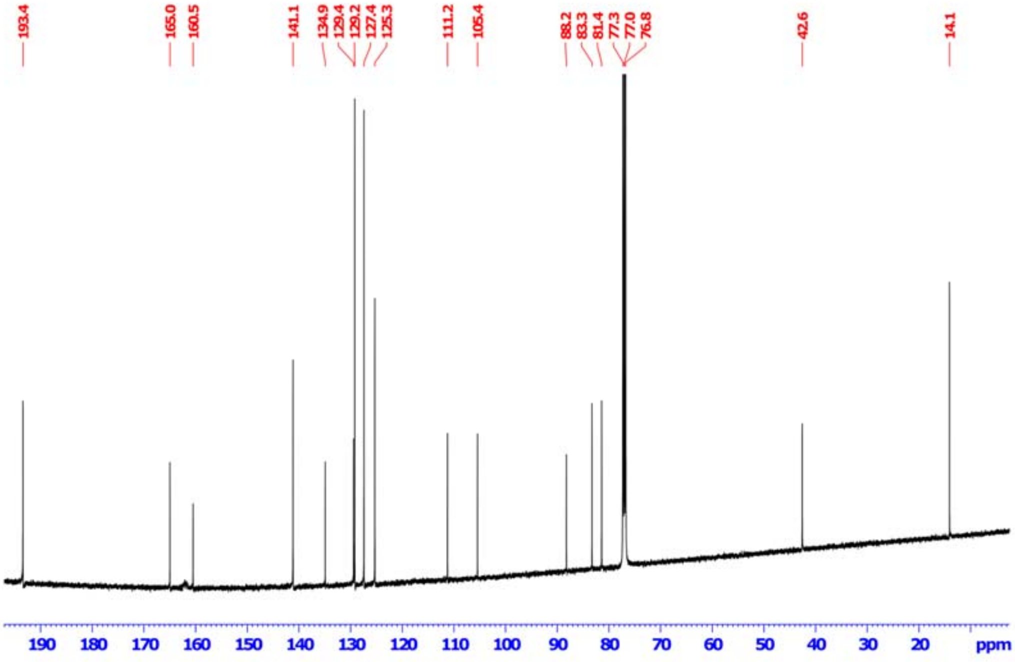

Figure S18: ^{13}C NMR of **6** in $CDCl_3$ (292 K).

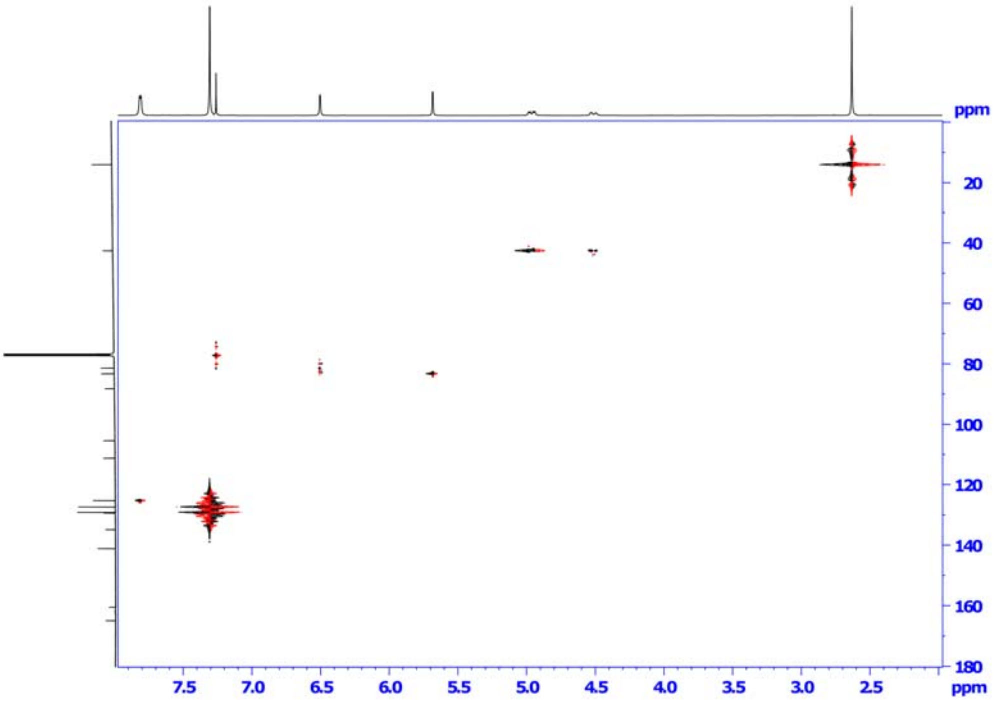

Figure S19: HSQC of **6** in $CDCl_3$ (292 K).

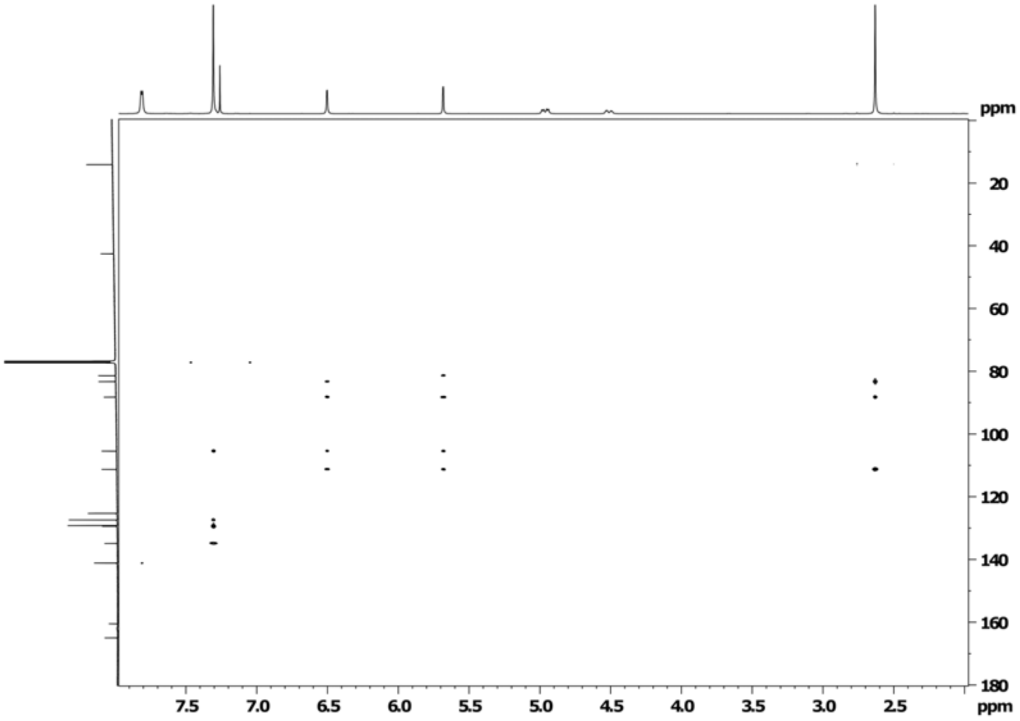

Figure S20: HMBC of **6** in $CDCl_3$ (292 K).

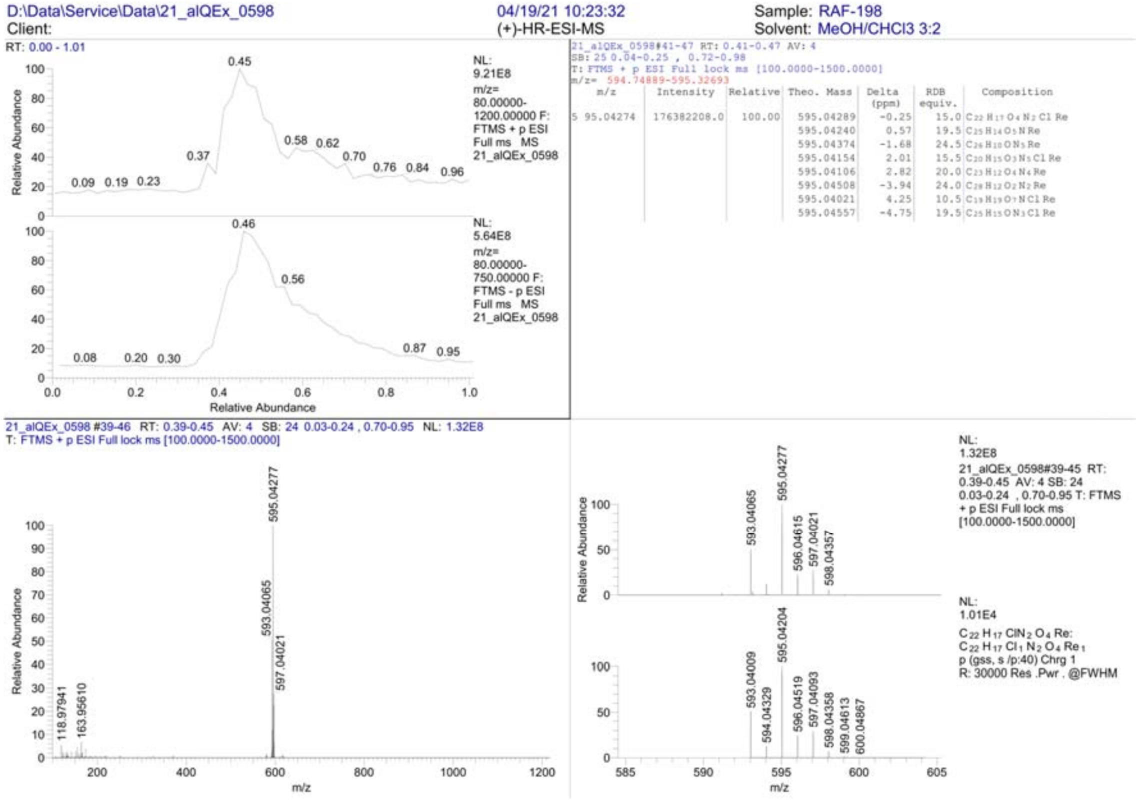

Figure S21: HR-ESI-MS of **6**.

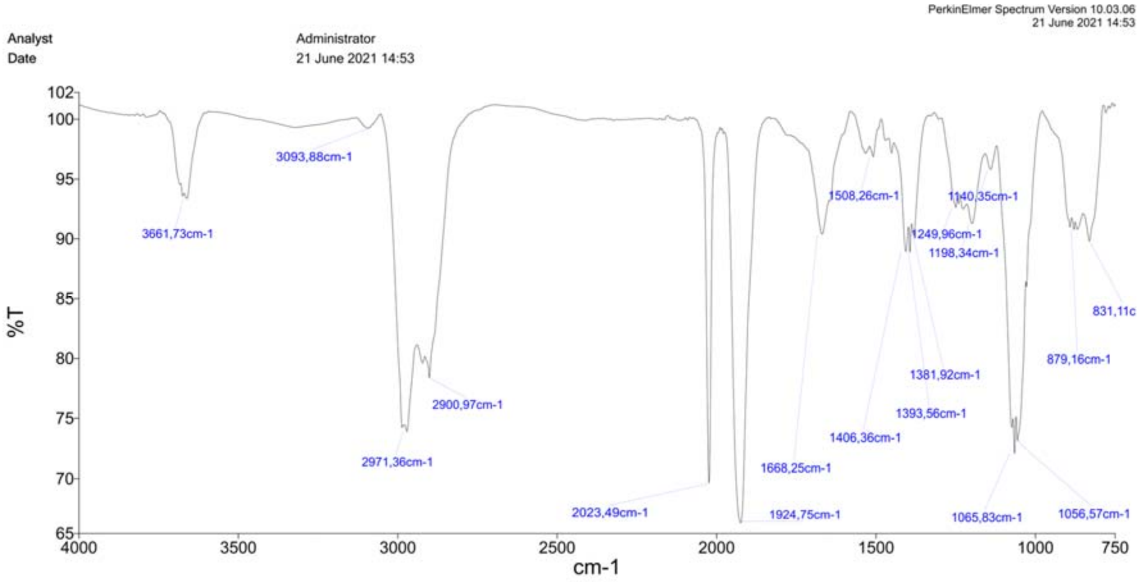

Figure S22: FT-IR of **6**.

6 Crystallographic Data

Table S3. Crystal data and structure refinement for **3.**

CCDC numer	2094783
Empirical formula	$C_{15}H_{15}ClO_2$
Formula weight	262.72
Temperature/K	160(1)
Crystal system	orthorhombic
Space group	$Pca2_1$
a/Å	25.9451(7)
b/Å	4.24250(10)
c/Å	11.7127(3)
α/°	90
β/°	90
γ/°	90
Volume/Å^3	1289.24(6)
Z	4
ρ_{calc}g/cm^3	1.354
μ/mm^{-1}	2.546
F(000)	552.0
Crystal size/mm^3	0.36 × 0.18 × 0.06
Radiation	Cu Kα (λ = 1.54184)
2Θ range for data collection/°	6.814 to 152.626
Index ranges	-32 ≤ h ≤ 32, -3 ≤ k ≤ 5, -14 ≤ l ≤ 14
Reflections collected	18917
Independent reflections	2682 [R_{int} = 0.0505, R_{sigma} = 0.0213]
Data/restraints/parameters	2682/1/165
Goodness-of-fit on F^2	1.160
Final R indexes [I>=2σ (I)]	R_1 = 0.0545, wR_2 = 0.1522
Final R indexes [all data]	R_1 = 0.0565, wR_2 = 0.1533
Largest diff. peak/hole / e Å^{-3}	0.42/-0.30

Flack parameter	0.000(17)

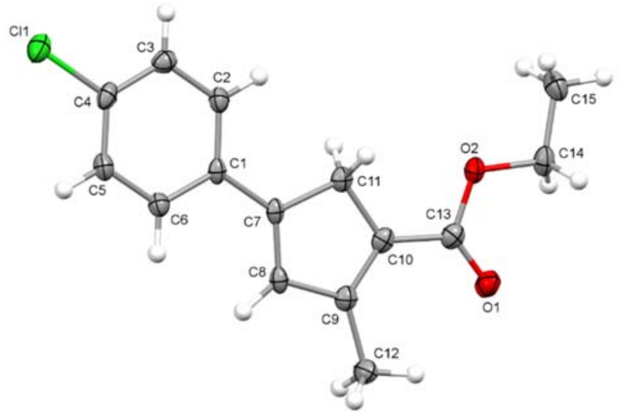

Figure S23. ORTEP representation of **3**. The displacement ellipsoids are represented at the 30% probability level.

Table S4. Crystal data and structure refinement for **4**.

CCDC number	2094782
Empirical formula	$C_{18}H_{14}ClO_5Re$
Formula weight	531.94
Temperature/K	160(1)
Crystal system	triclinic
Space group	P-1
a/Å	9.1670(2)
b/Å	9.36020(10)
c/Å	10.2239(2)
α/°	90.583(2)
β/°	103.616(2)
γ/°	90.8300(10)
Volume/$Å^3$	852.43(3)
Z	2
$\rho_{calc}g/cm^3$	2.072
μ/mm^{-1}	15.623
F(000)	508.0
Crystal size/mm^3	0.32 × 0.16 × 0.13
Radiation	Cu Kα (λ = 1.54184)
2Θ range for data collection/°	8.9 to 148.966
Index ranges	-11 ≤ h ≤ 11, -9 ≤ k ≤ 11, -12 ≤ l ≤ 12
Reflections collected	16835
Independent reflections	3480 [R_{int} = 0.0328, R_{sigma} = 0.0185]
Data/restraints/parameters	3480/0/229
Goodness-of-fit on F^2	1.184
Final R indexes [I>=2σ (I)]	R_1 = 0.0312, wR_2 = 0.0816
Final R indexes [all data]	R_1 = 0.0315, wR_2 = 0.0818
Largest diff. peak/hole / e $Å^{-3}$	2.63/-2.66

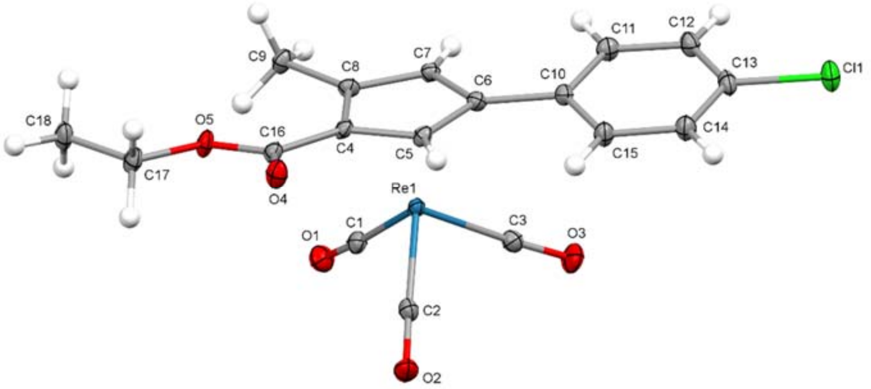

Figure S24. ORTEP representation of **4**. The displacement ellipsoids are represented at the 30% probability level.

Table S5. Crystal data and structure refinement for **6**.

CCDC number	2094784
Empirical formula	$C_{22}H_{16}ClN_2O_4Re$
Formula weight	594.02
Temperature/K	160(1)
Crystal system	monoclinic
Space group	$P2_1/n$
a/Å	8.39430(10)
b/Å	10.47450(10)
c/Å	23.0911(3)
α/°	90
β/°	95.3950(10)
γ/°	90
Volume/Å^3	2021.32(4)
Z	4
ρ_{calc}g/cm^3	1.952
μ/mm^{-1}	13.257
F(000)	1144.0
Crystal size/mm^3	0.49 × 0.37 × 0.14
Radiation	Cu Kα (λ = 1.54184)
2Θ range for data collection/°	7.692 to 148.982
Index ranges	-10 ≤ h ≤ 10, -12 ≤ k ≤ 13, -28 ≤ l ≤ 27
Reflections collected	20066
Independent reflections	4135 [R_{int} = 0.0282, R_{sigma} = 0.0147]
Data/restraints/parameters	4135/0/276
Goodness-of-fit on F^2	1.223
Final R indexes [I>=2σ (I)]	R_1 = 0.0210, wR_2 = 0.0534
Final R indexes [all data]	R_1 = 0.0211, wR_2 = 0.0534
Largest diff. peak/hole / e Å^{-3}	0.87/-0.61

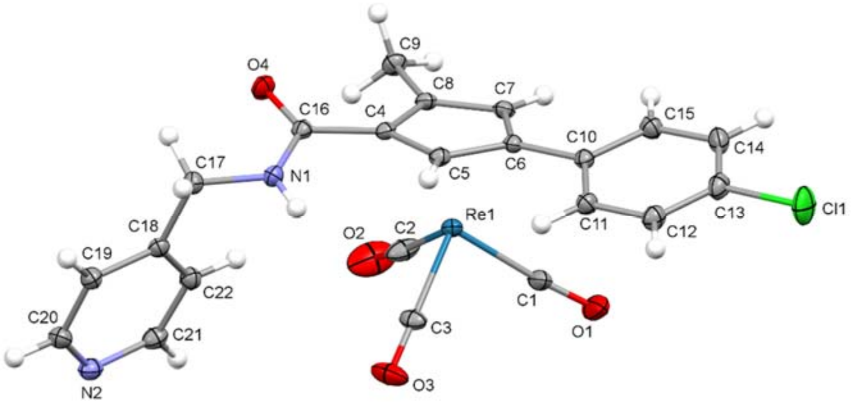

Figure S25. ORTEP representation of **6**. The displacement ellipsoids are represented at the 30% probability level.

References

(1) Clark, R. C.; Reid, J. S. *Acta Cryst. A* **1995**, 51, 887.

(2) *CrysAlisPro* (version 1.171.40.68a), Rigaku Oxford Diffraction Ltd, Yarnton, Oxfordshire, England, **2019**.

(3) "*A complete structure solution, refinement and analysis program*" - Dolomanov, O. V.; Bourhis, L. J.; Gildea, R. J.; Howard, J. A. K.; Puschmann, H. *J. Appl. Cryst.* **2009**, 42, 339.

(4) "*SHELXT - Integrated space-group and crystal-structure determination*" - Sheldrick, G. M. *Acta Cryst. A* **2015**, 71, 3.

(5) "*Crystal structure refinement with SHELXL*" - Sheldrick, G. M. *Acta Cryst. C* **2015**, 71, 3.

(6) "*Structure validation in chemical crystallography*" - Spek, A. L. *Acta Cryst. D* **2009**, *65*, 148.

(7) https://france.promega.com/resources/protocols/technical-manuals/0/adp-glo-kinase-assay-protocol/
